# Recovery sleep after total sleep deprivation preserves neutral and enhances emotional declarative memory

**DOI:** 10.1093/sleepadvances/zpaf093

**Published:** 2025-12-19

**Authors:** Tony J Cunningham, Dan Denis, Shengzi Zeng, Ryan Bottary, Elizabeth A Kensinger, Robert Stickgold

**Affiliations:** Center for Sleep and Cognition, Department of Psychiatry, Beth Israel Deaconess Medical Center, Boston, MA, United States; Division of Sleep Medicine, Harvard Medical School, Boston, MA, United States; Department of Psychology, University of York, York, United Kingdom; Center for Sleep and Cognition, Department of Psychiatry, Beth Israel Deaconess Medical Center, Boston, MA, United States; Division of Sleep Medicine, Harvard Medical School, Boston, MA, United States; Institute for Graduate Clinical Psychology, Widener University, Chester, PA, United States; Department of Psychology and Neuroscience, Boston College, Chestnut Hill, MA, United States; Center for Sleep and Cognition, Department of Psychiatry, Beth Israel Deaconess Medical Center, Boston, MA, United States; Division of Sleep Medicine, Harvard Medical School, Boston, MA, United States

**Keywords:** emotional memory, sleep deprivation, memory consolidation, recovery sleep, encoding

## Abstract

**Study Objectives:**

While recovery sleep can ameliorate the negative impacts of total sleep deprivation (TSD) on cognitive functioning, the effects of post-TSD sleep on different forms of emotional functioning remain unknown. Here, we investigated the effects of TSD and post-TSD recovery sleep on emotional memory processing.

**Methods:**

Participants viewed scenes with negative or neutral central objects overlain on neutral backgrounds. The scene components were then presented separately for recognition testing. Participants in the TSD (*n* = 46) and Sleep (*n* = 22) conditions encoded the scenes the morning after the sleep manipulation (~10:00) and recognition memory was tested for half of the scene components after a short delay (Recog_1, ~10:45). Twenty of the TSD participants then received a 90-min nap opportunity (TSD_Nap_). All participants then completed a second recognition test on the remaining images (Recog_2, ~14:00).

**Results:**

At Recog_1, all TSD participants showed worse overall memory compared to sleep participants. Specifically, memory was significantly worse for every scene component except neutral objects during Recog_1. At Recog_2, while memory deteriorated further for all scene components in the TSD_NoNap_ group, the TSD_Nap_ group showed no memory decline and had improved memory for negative objects, matching the sleep group at Recog_2.

**Conclusions:**

Post-TSD recovery sleep preserves and restores memory functioning to the level seen in typically rested individuals. But extending TSD leads to continued memory deterioration, highlighting the importance of sleep in healthy emotional memory functioning.

*This paper is part of the Festschrift in honor of Dr. Robert Stickgold*.

**Statement of Significance**

Napping after a night of total sleep deprivation (TSD) improves performance on many tasks. However, the impact that “recovery sleep” has on emotional memory has never been investigated prior to this study. As expected, total sleep loss prior to learning had a negative impact on all memory scores after a 10-min delay. TSD participants were then allowed a 90-min nap, or they stayed awake. At a second memory test, 4-h later, memory got worse for all stimuli in TSD participants that stayed awake. Intriguingly, the sleep deprived participants that napped showed no memory decline for neutral information and had *improved* memory for negative memory elements, resulting in memory abilities equivalent to those that slept normally the previous night.

## Introduction

A cornerstone of Dr. Robert Stickgold’s contribution to the field of sleep research is the progress that has been made in understanding the relationship between sleep and memory. His work has provided a fundamental piece of the puzzle, which has helped to establish that sleep almost unequivocally plays an important role in the consolidation of different types of memory [1–3]. However, while substantial evidence indicates periods of sleep generally benefit different forms of declarative and non-declarative (e.g. procedural) memories [4–7], investigations into whether sleep actively works to prioritize emotional memories over neutral memories continues to be fraught with mixed results and replication problems [8].

Two recent, independent meta-analyses perfectly illustrate this continued debate. The first meta-analysis from Lipinska and colleagues reported no overall effect for preferential sleep-dependent consolidation of emotional over neutral material across the published literature [9]. However, moderation analyses provided evidence for stronger effects when (1) studies used free recall rather than recognition outcome measures, and (2) when initial learning was controlled for in the delayed memory outcome measures. Further analyses suggested that other methodological features (e.g. (1) nocturnal sleep compared to daytime wakefulness, (2) nocturnal sleep compared to night-time sleep deprivation or restriction, or (3) a nap compared to wake study design) and sample characteristics may be confounding the effect of sleep on emotional memory processing. The second meta-analysis, from Schäfer et al., similarly failed to find a significant sleep-dependent prioritization of emotional over neutral material in memory processing [10]. The authors again highlighted that variability in methods, outcome measures, and analysis procedures used to investigate this effect likely contribute to the ambiguity in the field. Ultimately, both meta-analyses concluded that more fundamental, step-wise research needs to be done to clarify the strengths and limitations of this effect [11].

Beyond the simple comparison of sleep vs. wake, the inconsistency and uncertainty in the field also extends to work searching for the role of specific sleep stages associated with emotional memory consolidation. For decades, researchers have attempted to link the preferential processing of certain memory types to specific stages of sleep. Specifically, it has long been suggested that rapid eye movement (REM) sleep specifically supports emotional and procedural memory consolidation, while other stages of non-rapid eye movement (NREM) sleep enhances other forms of declarative memories [1, 2, 12, 13]. A comprehensive review by Davidson and colleagues highlights that the relegation of emotional memory processing strictly to REM sleep is likely not warranted [14], and it may be worth considering methods that go beyond the association of performance with minutes or percentage of sleep stages. To begin, a substantial majority (60+) of the datasets reviewed by Davidson et al. found no association of emotional memory performance with any sleep stage, and the ones that did find a correlation were mixed. For instance, an early paper by Wagner and colleagues found greater emotional memory benefits across late REM-rich sleep (as opposed to earlier SWS-rich sleep) and studies by Nishida et al., Payne et al., Groch et al., Wiesner et al., and Gilson et al. reported positive associations with measures of REM sleep and emotional memory performance [15–20]. However, not only have a number of studies similarly reported positive correlations of memory for emotional content with NREM measures of Stage 2 (N2) [21–25] and slow wave sleep (SWS) [25–30], but several studies have also reported negative correlations between emotional memory outcomes and measures of REM [24, 27, 31] and NREM [32, 33] sleep. Like the general comparison of sleep vs. wake, associating the preferential processing of emotional memories to a specific sleep stage continues to be plagued with mixed results and replication issues, highlighting a need to advance methods and analyses and take a more strategic approach to address the considerations that may influence these relationships [11].

Among these important considerations is the type (e.g. nap vs. overnight) and timing of sleep that occurs before, during, and after memory processing. To date, most of the research in this area has focused on sleep’s role during the early consolidation period of memory processing—shortly after encoding and before retrieval [11]. Sleep likely impacts all stages of memory consolidation and may affect the processing of emotional vs. neutral memories differently depending on the time that sleep or sleep loss is introduced. Another consideration is the type of memory task used to assess the preferential processing of aversive over neutral information. In an extensive review [8], the authors noted that the emotional memory trade-off (ETO) task appeared particularly adept at revealing preferential processing of emotional over neutral scene information during sleep.

In the ETO task, complex scenes are presented during encoding with negative or neutral central objects placed on an otherwise neutral background [34]. At recognition, the objects and backgrounds are presented separately and one at a time. While negative objects tend to be remembered better than their paired neutral backgrounds even after a short delay [34], numerous studies have indicated that a sleep-filled consolidation period amplifies the magnitude of this difference compared to a consolidation period of wakefulness [26, 35–37]. In this way, the ETO task is distinct from many other emotional memory tasks in that we are able to determine changes on different memory components within a single episode, which may be more ecologically relevant to our actual experience of emotional events. As such, this task may be the ideal tool to better understand the contexts in which sleep does and does not prioritize memory for negative information above and beyond concurrently learned neutral material.

Here, we used the ETO task to investigate the impact of total sleep deprivation (TSD) followed by recovery sleep on memory retention for negative and neutral scene components. This work adds to the field and to the legacy of Dr. Stickgold’s contributions in two novel ways. First, this work is the first to investigate the impact of sleep deprivation *prior* to the encoding of the ETO task.^1^ Second, while a substantial body of past evidence has indicated that post-TSD recovery sleep can restore typical functioning in a variety of basic cognitive tasks and measures [39–43], to our knowledge, this is the first investigation to look at the effect of recovery sleep on a cognitive task that includes an emotional component. As such, this study was designed to investigate the impacts of sleep and sleep loss both on the initial learning and the early consolidation phases of memory consolidation using this well validated emotional memory task.

In line with previous research that investigated the impact of sleep manipulation [38] and obstructive sleep apnea on ETO task performance [44], we predicted that TSD prior to encoding would generate significant initial impairment for all memory elements assessed, regardless of valence. We also predicted that a 90-min nap opportunity would prevent further deterioration in memory, such that those in the TSD group that were allowed a period of recovery sleep would perform significantly better on their post-nap assessment compared to sleep deprived participants that remained awake during the extended delay. Given the mixed findings highlighted above, the relatively small sample of participants with recorded polysomnography (PSG), and the expectation that the composition of the recovery sleep period after TSD would be substantially different than typical overnight sleep and nap periods (i.e. increased homeostatic sleep pressure and slow-wave activity), sleep stage and microarchitecture correlations were done in an exploratory manner.

## Materials and Methods

A total of 71 healthy participants (female = 54; 76%) were screened and enrolled into the study at Beth Israel Deaconess Medical Center (BIDMC). They were recruited from the greater Boston area through flyers and online job board postings. Eligibility criteria included age between 18 and 35 years (Range: 18-34 years, Mean = 21.96 years, SD = 3.78) and reported average of at least 6 h of sleep per night. Exclusion criteria included any antidepressant or sleep aid use, a sleep disorder or disability that impacts sleep, ongoing treatment for psychiatric or neurological disorders, learning or movement disorders, head trauma or concussions within the last two years, heart disease, vascular disease, or dementia, regular cigarette use, a history of substance or alcohol abuse/dependence. They also had to agree to abstain from alcohol and drug consumption from the day before the study through the end of the study. All participants provided informed consent; the study procedures were approved by the Institutional Review Board at BIDMC.

Participants were recruited into one of two conditions: a night of normal sleep (*n* = 22) or a night of TSD (n = 49). In the morning, TSD participants were further randomized into nap (TSD_Nap_) or no nap (TSD_NoNap_) conditions. Two TSD participants withdrew prior to nap group randomization, and ETO memory data from one TSD_Nap_ participant were lost due to technical error, leaving a total *N* = 68 (*n* = 22 normal sleep, *n* = 20 TSD_Nap_, *n* = 26 TSD_NoNap_). One participant in the TSD_Nap_ condition did not complete the morning self-report assessments. Further demographics are reported in Table 1. Data collection began in May 2019. Given the substantial impact that TSD has on memory encoding [45], a power analysis using G^*^Power (version 3.1.9.7) [46] indicates that a sample size of 20 would be sufficient to achieve 90 % power (given an alpha of 0.05) to detect the negative impact of TSD on memory. We targeted 25 per group in an attempt to better power the exploratory sleep stage analyses in the TSD_nap_ condition. In March of 2020, data collection was interrupted by the COVID-19 pandemic. After research restrictions were lifted, we exhausted the remaining funding we had, collecting as many additional subjects as we could afford. As such, resource constraints was a significant contributor to the final group sizes [47].

**Table 1:** Demographic and assessment data

	Normal sleep (*n* = 22)	TSD_Nap_ (*n* = 20)	TSD_NoNap_ (*n* = 26)
Age (years)	23.0 ± 4.0	21.7 ± 3.7	20.9 ± 2.9
Female sex (*n*, %)	16 (72.7%)	18 (90.0%)	18 (69.2%)
Gender			
Male	5 (22.7%)	2 (10.0%)	8 (30.8%)
Female	15 (68.2%)	17 (85.0%)	18 (69.2%)
Self-described	1 (4.5%)	0	0
Not reported	1 (4.5%)	1 (5.0%)	0
Race			
American Indian/Alaska Native	0	0	0
Asian	7 (31.8%)	9 (45.0%)	8 (30.8%)
Black	3 (13.6%)	1 (5.0%)	6 (23.1%)
Native American or Other Pacific Islander	0	0	0
White	9 (40.9%)	9 (45.0%)	8 (30.8%)
More than one race	3 (13.6%)	1 (5.0%)	0
Unknown/not reported	0	0	4 (8.7%)
Ethnicity			
Hispanic	7 (31.8%)	3 (15.0%)	5 (19.2%)
Not Hispanic	15 (68.2%)	17 (85.0%)	20 (76.9%)
Unknown/not reported	0	0	1 (3.8%)
	**Normal Sleep (M ± SD)**	**TSD_Nap_ (M ± SD)**	**TSD_NoNap_ (M ± SD)**
Sleep (h)	6.3 ± 0.9^a^	–	–
ESS	7.1 ± 3.6	7.5 ± 3.6	7.1 ± 3.6
BDI-II	8.4 ± 8.66	6.1 ± 4.5	9.2 ± 7.3
PSQI	3.6 ± 2.1	3.9 ± 1.9	4.0 ± 1.9
PA_Baseline	**33.4 ± 7.2**	31.0 ± 7.9	**27.7 ± 7.4**
PA_Morning	**29.0 ± 9.9**	**17.5 ± 7.6** ^**b**^	**15.4 ± 5.0**
PA_Afternoon	**27.0 ± 9.7**	**20.6 ± 9.1**	**19.6 ± 9.1**
NA_Baseline	13.9 ± 5.4	13.7 ± 2.8	13.9 ± 2.7
NA_Morning	11.2 ± 2.3	11.5 ± 2.2^b^	13.3 ± 4.8
NA_Afternoon	11.7 ± 3.1	11.1 ± 1.8	11.8 ± 2.1
SSS_Baseline	2.6 ± 0.8	2.7 ± 1.1	2.9 ± 0.9
SSS_Morning	**2.4 ± 1.2**	**4.6 ± 1.5** ^**b**^	**5.0 ± 1.1**
SSS_Afternoon	**2.4 ± 1.5**	3.1 ± 1.4	**4.0 ± 1.8**
Refreshed_Baseline	57.6 ± 26.8	55.5 ± 27.9	55.2 ± 20.2
Refreshed_Morning	**63.6 ± 29.8**	**29.8 ± 21.7** ^**b**^	**24.6 ± 21.3**
Refreshed_Afternoon	**65.4 ± 17.9**	55.5 ± 30.4^*^	33.1 ± 27.3^*^
Concentrate_Baseline	72.2 ± 21.1	74.2 ± 24.8	67.9 ± 19.6
Concentrate_Morning	**70.4 ± 23.2**	**33.8 ± 25.1** ^**b**^	**22.3 ± 19.7**
Concentrate_Afternoon	**72.0 ± 19.7**	**57.25 ± 27.2**	**41.6 ± 28.0**
PVT Omissions_Baseline (%)	6.8 ± 9.1	5.8 ± 7.2	7.8 ± 9.4
PVT Omissions_Morning (%)	**4.7 ± 6.4**	**11.4 ± 16.4**	**13.3 ± 15.8**
PVT Omissions_Afternoon (%)	3.5 ± 4.6	5.3 ± 6.7	7.3 ± 13.2

Demographic and assessment scores for the Normal Sleep, TSD nap, and TSD no nap conditions. Sleep, number of hours of self-reported sleep in the Normal Sleep control condition the night of the sleep manipulation; ESS, Epworth Sleepiness Scale; BDI-II, Beck Depression Inventory, 2^nd^ edition; PSQI, Pittsburgh Sleep Quality Index; PA, Positive Affect; NA, Negative Affect; SSS, Stanford Sleepiness Scale; PVT, Psychomotor Vigilance_task; PVT Omission, reaction time > 500 ms; Baseline, 10 p.m. pre-sleep manipulation; Morning, 09:30 post-sleep manipulation; Afternoon, 13:30 post-sleep manipulation; M, mean; SD, standard deviation. ^a^Two Normal Sleep participants did not fill out the morning sleep log due to experimenter error, leaving n = 20. ^b^One TSD_Nap_ participant did not complete the morning self-report assessments, leaving *n* = 19. Bolded means indicate significant differences between the Normal Sleep control condition and one or both TSD groups as indicated (*p* < .05). ^*^Indicate a significant difference between the TSD_Nap_ and TSD_NoNap_ groups (*p* < .05).

### Study procedures

On the evening of their study visit, participants were asked to arrive between 20:30 and 21:00 and completed a battery of assessments, including the Epworth Sleepiness Scale [48], Beck Depression Inventory, 2^nd^ Edition [49], Pittsburgh Sleep Quality Index [50], and baseline measures of the Positive and Negative Affect Schedule (PANAS) [51] and the Stanford Sleepiness Scale (SSS) [52]. Participants also gave baseline ratings (0–100) of their ability to concentrate and how refreshed they were feeling in the moment, as well as performing a 3-min version of the Psychomotor Vigilance task (PVT) as a baseline measure of attention and reaction time [53].

After the evening battery of assessments and cognitive tasks, participants in the normal sleep group were sent home to sleep (typically leaving between 22:30 and 23:00) and were asked to return at 08:30 a.m. after sleeping normally at home (Table 1). Participants in the TSD condition were set up for a night of observed sleep deprivation in the Clinical Research Center at BIDMC. Participants were allowed to do homework, watch TV/movies, play games, and were under constant supervision of a study staff member to ensure adherence to the protocol. In the morning, all participants were given breakfast (~08:30) and completed another battery of cognitive and clinical assessments, including reassessment of the PANAS, SSS, ratings of concentration and refreshedness, and the PVT.

They then completed the encoding session of the ETO task [34] (see below). After a 10-min delay (during which participants completed additional questionnaires), participants completed the first recognition session of the ETO task (Recog_1). After the memory test, participants in the TSD group were further randomized into nap (TSD_Nap_) and no nap (TSD_NoNap_) conditions. After randomization, all participants were provided lunch and participants in the TSD_Nap_ condition received a 90-minute nap opportunity with high-density PSG recording (see section PSG acquisition and preprocessing). One nap TSD_Nap_ participant slept without PSG due to an equipment issue at the time of participation. Participants in the TSD_NoNap_ and normal sleep conditions remained awake during the nap period and were again constantly monitored to ensure adherence to the protocol.

**Figure 1:**
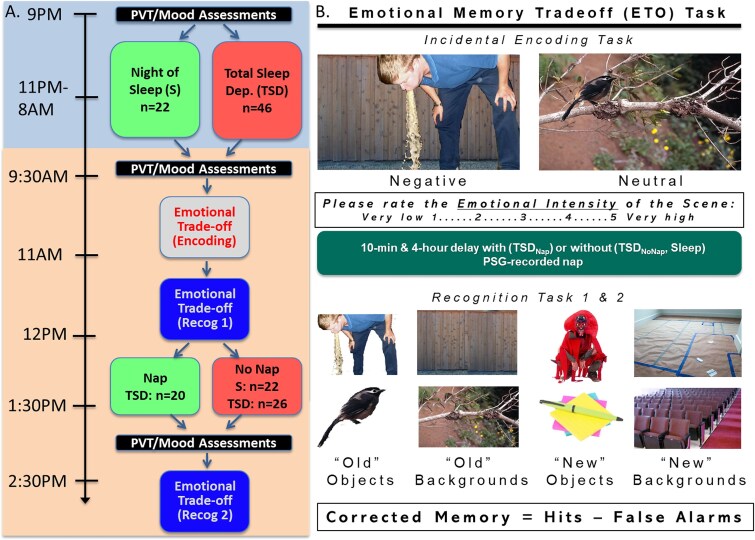
(A) Study timeline (B) sample stimuli used during the encoding and recognition sessions of the emotional memory trade-off (ETO) paradigm.

After the nap period, participants in the TSD_Nap_ condition were awakened, after which all participants followed identical protocols for the remainder of the study. To allow for dissipation of sleep inertia in the TSD_Nap_ condition, all participants began this session with another round of assessments, including the PANAS, SSS, ratings of concentration and refreshedness, and PVT. They then completed a second protocol with a recognition task on the remaining ETO images (see Figure 1A). Upon completion of the study, participants were debriefed, compensated, and dismissed. For safety, all TSD participants were provided with a free ride home using ridesharing services.

### Emotional trade-off task

Participants completed the ETO task [34], a task of emotional memory with demonstrated sensitivity to assess sleep-dependent emotional memory consolidation [8, 18, 26, 36, 37, 54]. During the encoding session, participants studied 104 previously validated scenes [34]—52 with neutral objects (e.g. an avocado) on neutral backgrounds (e.g. a kitchen counter) and 52 with negatively arousing objects (e.g. a severed foot) on different neutral backgrounds (e.g. a living room floor; Figure 1B). Participants were shown one of two different sets of images to control for potential list effects. Each scene was displayed for 4000 ms on a laptop computer screen and, once removed, participants had 4000 ms to respond to a prompt assessing the perceived emotional intensity of the scene on a scale of 1 (very low) to 5 (very high). During both recognition tasks, objects and backgrounds were presented separately and one at a time, in random order. Foils were presented at ~70% ratio compared to old images. As such, each recognition task included 52 same objects (26 negative and 26 neutral), 52 same backgrounds (26 previously shown with a negative object and 26 shown with a neutral object), 36 new objects (18 negative and 18 neutral), and 36 new neutral backgrounds (see Figure 1B). Participants were asked to identify each object and background presented as “Old” or “New.”

### PSG acquisition and preprocessing

For participants in TSD_NoNap_ condition, their nap electroencephalography (EEG) was recorded from 57 scalp electrodes arranged according to the international 10–20 system. Additional electrodes were positioned at the left and right mastoids, above the right eye and below the left eye (electrooculogram), on the chin (electromyogram), on the forehead (reference), and on the collarbone (ground). Data were collected using an Aura-LTM64 amplifier with TWin software (Grass Technologies), at a sampling rate of 400 Hz, with all electrode impedances targeted below 25 kΩ. There were 20 participants in the TSD_nap_ condition. One participant did not sleep with PSG due to an equipment issue at the time of participation, one participant’s PSG data did not save correctly, and two participants were excluded due to poor EEG data quality, leaving *n* = 16 nap PSGs for analysis.

Nap EEG was exported as European Data Format files for further offline preprocessing and analysis using EEGLAB/ERPLAB [55, 56], and Danalyzer toolboxes [57] in MATLAB (MathWorks, Natick, MA). Offline preprocessing included bandpass filters (0.3–35 Hz for EEG, 10–100 Hz for EMG) and a 60 Hz notch filter. A trained study staff (S.Z.) performed sleep scoring following American Academy of Sleep Medicine (AASM) Scoring Manual Version 2.6 [58]. Extracted sleep metrics included time in bed (TIB), total sleep time (TST), sleep onset latency, wake after sleep onset (WASO), sleep efficiency (SE), and time spent in N1/N2/N3/REM (in minutes and percentage).

EEG artifacts were first identified using an automated algorithm and then verified through visual inspection. For each channel, Hjorth parameters (activity, mobility, complexity [59]) were calculated for every epoch. Bad channels were flagged if more than 50% of their epochs exceeded 3 standard deviations from the mean on any of the three parameters. Likewise, epochs were marked as artifacts if at least 17 channels within that epoch exceeded 3 standard deviations from the mean on any of the three parameters. These flagged channels and epochs were visually reviewed. Confirmed bad channels were interpolated using a spherical spline method [57]. Artifactual epochs were excluded from subsequent analyses.

Power spectral density (PSD) was estimated separately for NREM (stages N2/N3) and REM sleep at frontal (Fz, F1, F2, F3, F4), central (Cz, C1, C2, C3, C4), and posterior (Pz, P1, P2, P3, P4) electrodes. Using Welch’s method with 5-s Hamming windows and 50% overlap, PSD was derived from the first derivative of the EEG signal to minimize 1/f effects [60]. PSD values from 0 to 30 Hz were normalized within participants by dividing each frequency bin by the mean power across this range [61]. Values were then averaged across frontal, central, and posterior sites, yielding composite measures of regional PSD activity.

Spindles were identified during NREM sleep with a wavelet-based detector [57, 62, 63]. A spindle was defined as a 12–15 Hz event with power exceeding six times the median signal amplitude for ≥400 ms. Extracted features included frequency, density, amplitude, and duration. Slow oscillations were detected by band-pass filtering signals at 0.5–4 Hz and identifying successive zero-crossings within 0.5–2 s. Events in the top quartile of peak-to-peak amplitude were retained as slow oscillations [64].

Coupling was assessed by filtering the signal in delta (0.5–4 Hz) and spindle (12–15 Hz) bands, extracting instantaneous phase and amplitude with the Hilbert transform. For each spindle, its peak amplitude was tested for co-occurrence within a slow oscillation. When present, the spindle peak phase angle relative to the slow oscillation was computed. Coupling metrics included the proportion of coupled spindles, densities of coupled vs. uncoupled events, mean phase angle, and coupling strength (vector length [57]). These measures were then related to memory outcomes.

### Statistical analysis

ETO memory performance was calculated separately for each valence (negative and neutral) and scene component (objects and backgrounds). The magnitude of the object-background trade-off was calculated as the difference between object and background memory scores within the same valence (e.g. negative trade-off = memory for negative objects minus memory for their paired backgrounds). Uncorrected memory scores were calculated as the total correct responses divided by the total number of images for each stimulus type. To correct for response bias, we calculated corrected memory scores by subtracting the proportion of false alarms (i.e. “Old” judgments to new pictures) of each valence and scene component type from the proportion of hits [65]. Repeated measures ANOVAs of Condition x Scene Component x Valence were used for initial memory tests and were then followed up by t-tests when appropriate. Demographic and assessment variables were analyzed using chi-square (categorical) and ANOVA (continuous) assessments with follow-up *t*-tests as indicated. For significant ANOVAs related to the ETO task, object-background and negative-neutral comparisons were planned a priori. An alpha level of 0.05 was used to determine statistical significance. Cohen’s *d* and partial eta square effect sizes are reported throughout.

## Results

### Demographics and baseline self-report metrics

Demographic and all baseline self-report assessments revealed no significant demographic (age, sex, gender, race, ethnicity) or baseline differences between the three conditions (normal sleep, TSD_Nap_, TSD_NoNap_) on the evening prior to the sleep manipulation (all *p*’s > .10), except the participants that would go on to be randomized into the TSD_NoNap_ condition the next morning had significantly lower positive affect (PA) compared to participants in the normal sleep condition (*t*(46) = 2.7, *p* = .01, *d* = 0.78). However, participants in the TSD_Nap_ and TSD_NoNap_ conditions did not differ in PA at baseline (*p* = .16).

### Psychomotor vigilance task

Errors of omission on the PVT (typically defined as reaction times >500 ms) have been demonstrated to be the most sensitive metric to sleep loss [66–68] (Table 1). Using the standard 500 ms cutoff, there were no differences between the three conditions during the evening baseline session (*p* = .74). In the morning, participants in the TSD conditions had significantly more omission errors than normal sleep participants (*F*_1,66_ = 4.76, *p* = .03, η^2^ = 0.20) demonstrating the detrimental effects of sleep loss on vigilance and attention. Notably, there was no difference between the TSD_NoNap_ and TSD_Nap_ participants prior to randomization (*p* = .67). In the afternoon, PVT performance returned to baseline levels in both TSD conditions leading to no difference between the three conditions (*p* = .37). This restoration of performance in the afternoon following sleep deprivation is in line with previous demonstrations of the influence of circadian rhythms on vigilance and attention [69, 70].

### Emotional trade-off task: 10-min delay (Recog_1)

We first determined the effects of sleep deprivation on ETO memory processing after the 10-min post-training delay (Recog_1). To determine if there was an effect of sleep deprivation, we ran a 2 (group: sleep, TSD) × 2 (scene component: object, background) × 2 (object valence: negative, neutral) ANOVA, with scene component and valence as repeated measures [34]. This analysis revealed a main effect of scene component (objects vs. backgrounds; *F*_1,66_ = 46.07, *p* < .001, η^2^ = 0.41), and a two-way interaction between scene component and valence (negative vs. neutral; *F*_1,66_ = 59.43, *p* < .001, η^2^ = 0.47), which together represent the ETO effect [34]. Specifically, negative objects were significantly better remembered than neutral objects (*t*(67) = 6.68, *p* < .001, *d* = 0.81), and memory performance was significantly worse for backgrounds paired with negative objects compared to backgrounds paired with neutral objects (*t*(67) = 6.27, *p* < .001, *d* = 0.76). Additionally, negative objects were remembered significantly better than their paired backgrounds (*t*(67) = 11.78, *p* < .001, *d* = 1.43), while memory for neutral objects and their paired backgrounds was statistically similar (*p* = .69).

**Figure 2:**
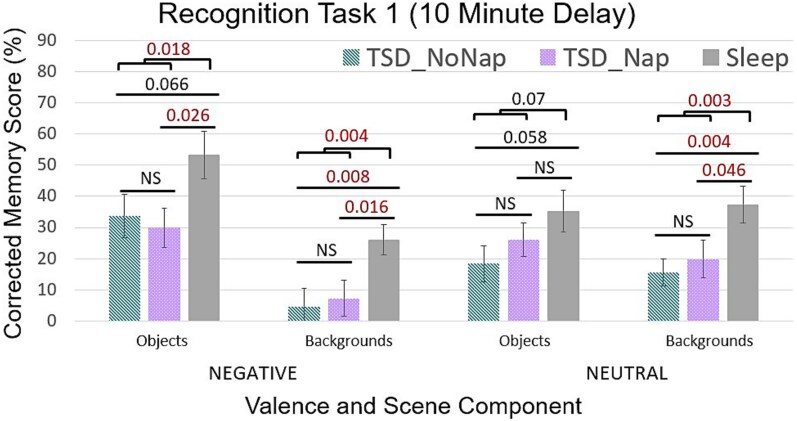
ETO memory results for participants in the sleep and TSD conditions at Recog_1 (10-minute delay, prior to the nap/no-nap differentiation). Compared to the sleep condition, TSD participants had significant impairments in all measures of corrected recognition memory except neutral objects, which failed to reach significance. Participants that would go on to be randomized into the TSD_Nap_ and TSD_NoNap_ conditions did differ at Recog_1. Error bars = SEM. Significant *p*-value comparisons < 0.05. NS = not significant.

In support of our first hypothesis, there was also a main effect of group (sleep vs. TSD; *F*_1,66_ = 7.88, *p* = .007, η^2^ = 0.11). Follow up analyses revealed that this main effect was driven by poorer memory following TSD compared to sleep for all scene elements except neutral objects (*p* = .07) (negative objects: *t*(66) = 2.43, *p* = .02, *d* = 0.63; backgrounds paired with negative objects: *t*(66) = 2.99, *p* = .004, *d* = 0.78; backgrounds paired with neutral objects: *t*(66) = 3.04, *p* = .002, *d* = 0.79). Again, there were no baseline differences between TSD_Nap_ and TSD_NoNap_ participants in memory performance for any scene element prior to randomization (all *p*’s > .34; see Figure 2).

### Emotional trade-off task: 4 h delay (Recog_2)

To determine the impact of continued sleep deprivation vs. a period of recovery sleep on ETO memory performance, we conducted a 3 (condition: sleep, TSD_NoNap_, TSD_Nap_) × 2 (scene component: object, background) × 2 (object valence: negative, neutral) ANOVA, with scene component and valence as repeated measures on ETO memory after the 4-h delay (Recog_2). Overall, the ETO effect continued to persist as indicated by a main effect of scene component (objects vs. backgrounds; *F*_1,65_ = 79.15, *p* < .001, η^2^ = 0.55), and a two-way interaction between scene component and valence (negative vs. neutral; *F*_2,65_ = 54.76, *p* < .001, η^2^ = 0.46). There was also a main effect of valence indicating that at the 4-h delay negative scene components (objects and backgrounds) were overall remembered better than neutral scene components (*F*_1,65_ = 4.26, *p* = .04, η^2^ = 0.06). The repeated measures ANOVA at Recog_2 also revealed a main effect of condition (*F*_2,65_ = 11.13, *p* < .001, η^2^ = 0.26).

**Figure 3:**
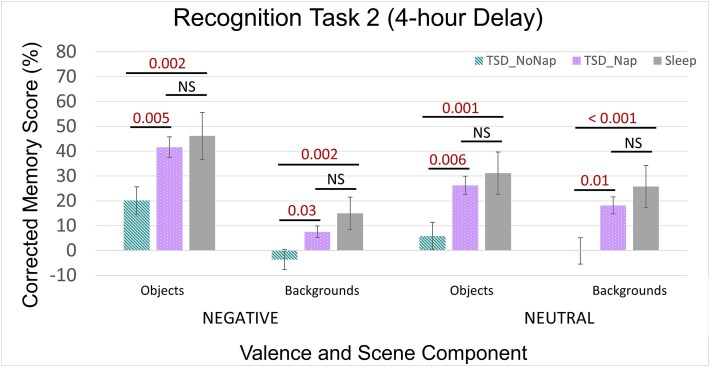
ETO memory results for sleep, TSD_Nap_, and TSD_NoNap_ participants at Recog_2 (4-h delay). TSD_NoNap_ participants had significant impairments in all aspects of corrected recognition memory compared to both the sleep and TSD_Nap_ conditions, while TSD_Nap_ participants no longer differed from the sleep control group after the extended delay. Error bars = SEM. Significant *p*-value comparisons < 0.05. NS = not significant.

*Post hoc* analyses revealed that participants in the Sleep condition performed significantly better than those in the TSD_NoNap_ condition for all scene component types (negative objects: *t*(46) = 3.37, *p* = .002, *d* = 0.98; backgrounds paired with negative objects: *t*(46) = 3.37, *p* = .002, *d* = 0.98; neutral objects: *t*(46) = 3.38, *p* = .001, *d* = 0.98; backgrounds paired with neutral objects: *t*(46) = 3.55, *p* < .001, *d* = 1.03). Participants in the TSD_Nap_ group, however, showed no statistically significant differences in performance compared to those in the sleep condition for all scene component types following their nap (all *p*’s > .10), indicating that the period of recovery sleep after TSD restored memory performance to what would be expected in a typically rested state. Moreover, in support of our second hypothesis TSD_Nap_ participants performed significantly better than TSD_NoNap_ participants on all valence and scene component scores at Recog_2 (negative objects: t(44) = 2.96, *p* = .005, *d* = 0.88; backgrounds paired with negative objects: *t*(44) = 2.22, *p* = .03, *d* = 0.66; neutral objects: *t*(44) = 2.86, *p* = .006, *d* = 0.85; backgrounds paired with neutral objects: *t*(44) = 2.70, *p* = .01, *d* = 0.80; see Figure 3).

### Emotional trade-off task: change from Recog_1 to Recog_2

**Figure 4:**
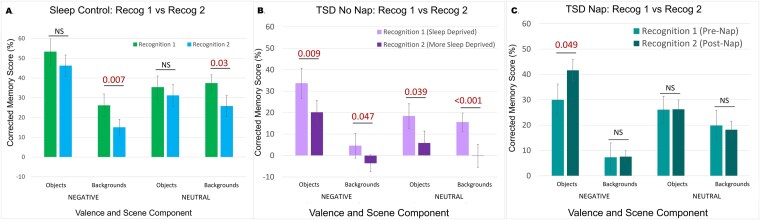
These figures re-plot the data from Figures 2 and 3 to show the change in ETO memory performance from Recog_1 to Recog_2 for all groups. (A) Participants in the sleep condition showed significant deterioration in memory for peripheral neutral background memory, (B) TSD_NoNap_ participants showed significant deterioration in memory for all scene components, regardless of valence, and (C) TSD_Nap_ participants showed no deterioration for any neutral scene components and a significant improvement in memory for negative central objects from Recog_1 to Recog_2. Error bars = SEM. Significant *p*-value comparisons < 0.05. NS = not significant.

**Figure 5:**
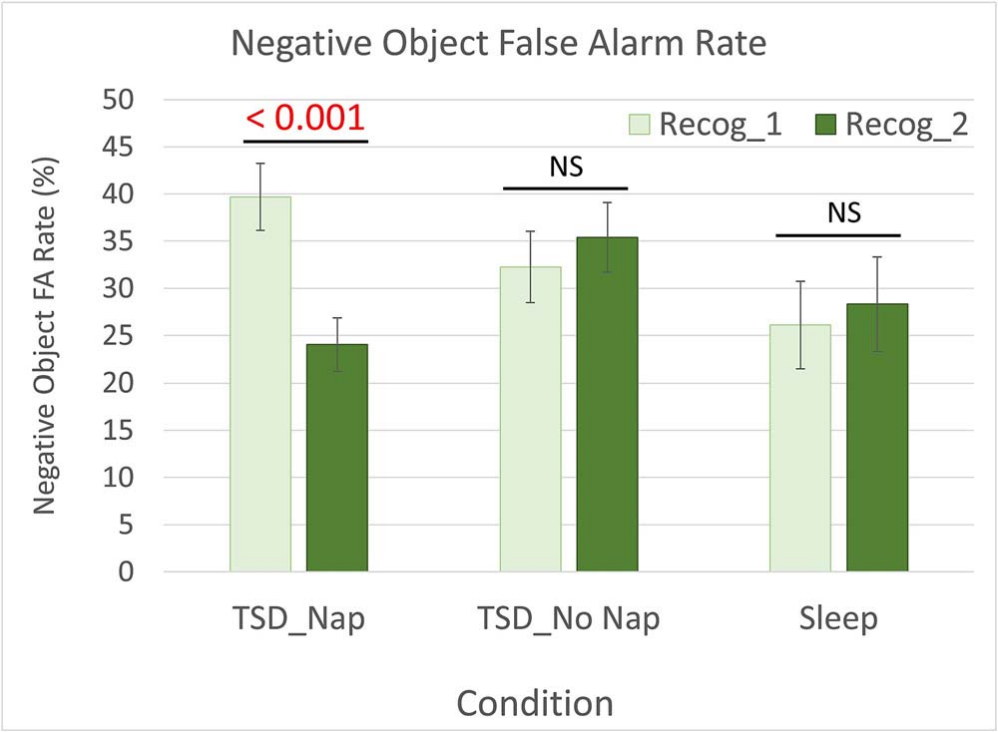
The negative object false alarm rate (i.e. identifying “new” negative objects as “old”) significantly reduced from pre- to post-nap testing sessions (Recog_1 to Recog_2) in TSD_Nap_ participants. There was no change in negative false alarm rate from Recog_1 to Recog_2 in either the TSD_NoNap_ or sleep conditions. Error bars = SEM. Significant *p*-value comparisons < 0.05. NS = not significant.

After demonstrating that (1) TSD prior to encoding had largely negative effects on ETO task memory performance after a short delay, (2) the ETO effect persisted in all groups for both recognition sessions, and (3) that a brief period of recovery sleep restored ETO memory function to typically rested levels while continued sleep deprivation led to further deterioration, we then conducted comparisons to see how performance changed within each group from Recog_1 to Recog_2 (see Table 2). Participants in the Sleep condition retained memory for negative and neutral objects but had significant deterioration for neutral backgrounds—regardless of object valence—from Recog_1 to Recog_2, demonstrating some level of normal deterioration for neutral peripheral details in a rested state (see Figure 4A). The TSD_NoNap_ group showed continued deterioration in all scene component and valence types, demonstrating the devastating impact of continued total sleep loss on memory, with only negative objects showing any significant memory retention (*t*(25) = 3.69, *p* = .001, *d* = 0.72; see Figure 4B). The TSD_Nap_ group, however, while showing no significant change in memory for either neutral scene component (i.e. neutral objects or backgrounds paired with neutral objects) or for backgrounds paired with negative objects, showed significant *improvement* in memory for negative objects from Recog_1 to Recog_2, demonstrating an unexpected enhancement in memory for negative information following recovery sleep (see Figure 4C). Given that these were corrected memory scores (i.e. hits—false alarms), this effect on negative object memory could have been driven by: (1) an increase in correct memory, (2) a decrease in false alarms, or (3) a combination of both. But *post hoc* analyses revealed that this effect was largely driven by a significant decrease in false alarms from Recog_1 to Recog_2, *t*(19) = 3.96, *p* < .001, *d* = 0.89 (see Figure 5).

**Table 2:** Within-group comparisons of corrected memory scores

Sleep control participants							
	**Recog**_**1 (M ± SE)**	**Recog**_**2 (M ± SE)**	**Mean difference**	**95% CI lower**	**95% CI upper**	***t*-Score**	***P*-value**
Negative objects	53.2 ± 7.7	46.1 ± 5.3	7.1	−6.3	20.5	1.11	.28
Backgrounds paired with negative objects	26.1 ± 4.9	15.0 ± 3.7	11.1	3.4	18.8	3.01	.007^*^
Neutral objects	35.3 ± 6.6	31.1 ± 4.8	4.2	−4.9	13.3	0.95	.36
Backgrounds paired with neutral objects	37.4 ± 5.6	25.7 ± 4.8	11.6	1.5	21.7	2.39	.03^*^
**TSD no nap participants**							
	**Recog**_**1 (M ± SE)**	**Recog**_**2 (M ± SE)**	**Mean difference**	**95% CI lower**	**95% CI upper**	***t*-Score**	***P*-value**
Negative objects	33.7 ± 7.0	20.2 ± 5.5	13.5	3.7	23.3	2.84	.009^*^
Backgrounds paired with negative objects	4.6 ± 5.8	−3.6 ± 4.0	8.2	0.1	16.2	2.09	.047^*^
Neutral objects	18.4 ± 5.7	5.8 ± 5.6	12.6	0.7	24.5	2.18	.039^*^
Backgrounds paired with neutral objects	15.5 ± 4.3	−0.1 ± 5.3	15.7	8.6	22.7	4.57	<.001^*^
**TSD nap participants**							
	**Recog_1 (M ± SE)**	**Recog_2 (M ± SE)**	**Mean difference**	**95% CI lower**	**95% CI upper**	***t*-Score**	***P*-value**
Negative objects	29.9 ± 6.3	41.6 ± 4.2	−11.7	−23.3	−0.07	2.11	.049^*^
Backgrounds paired with negative objects	7.3 ± 5.7	7.6 ± 2.3	−0.3	−13.8	13.2	0.05	.97
Neutral objects	26.0 ± 5.3	26.2 ± 3.7	−0.2	−10.2	9.8	0.04	.97
Backgrounds paired with neutral objects	19.8 ± 6.1	18.1 ± 3.4	1.6	−11.5	14.8	0.26	.80

Within-group comparisons of corrected memory scores for each valence and scene type for each group (Sleep, TSD_Nap_, TSD_NoNap_). Mean ± SEM, mean difference, and 95% confidence interval (CI) reported. Comparison between groups done using paired-samples *t*-tests

^^*^^Significance set at *p* ≤ .05.

### Object-background difference scores

Remarkably, while overall memory performance (i.e. the number of items correctly remembered) differed dramatically between conditions and across sessions, each condition maintained a similar pattern with regard to the difference in memory for the negative scene components compared to the difference in memory for the neutral scene components at both the 10-min and 4-h recognition tasks. Specifically, all conditions—regardless of sleep amount or recovery sleep opportunity—demonstrated significantly better retention for negative objects compared to their paired backgrounds at both recognition sessions, while memory for neutral objects and their paired backgrounds did not differ. In addition, for all conditions, the difference in memory for negative objects and their paired backgrounds was significantly greater than the difference between neutral objects and their matched backgrounds at each recognition session (see Table 3). Moreover, the magnitude of these negative and neutral object-background differences did not differ between any conditions at Recog_1 or at Recog_2 (all *p*’s > .13). This means that—despite significant deterioration in memory overall—the ETO effect persists even after a substantial period of TSD.

**Table 3:** Within-group comparison of object-background difference scores

**Within-group object-background difference comparisons**							
**Sleep control participants**	**Negative scene object-background Difference (M ± SE)**	**Neutral scene object-background Difference (M ± SE)**	**Negative − neutral scene difference**	**95% CI lower**	**95% CI upper**	***t*-Score**	***P*-value**
Recognition 1 (10-min delay)	27.2 ± 4.5	−2.1 ± 2.9	29.3	19.1	39.4	5.98	<.001^*^
Recognition 2 (4-h delay)	31.2 ± 4.1	5.4 ± 3.8	25.8	15.7	35.6	5.46	<.001^*^
**TSD nap participants**	**Negative Scene Object-Background Difference (M ± SE)**	**Neutral Scene Object-Background Difference (M ± SE)**	**Negative − Neutral Scene Difference**	**95% CI Lower**	**95% CI Upper**	***t*-Score**	***P*-value**
Recognition 1 (10-min delay)	22.7 ± 3.8	6.2 ± 6.6	16.4	2.8	30.1	2.52	.02^*^
Recognition 2 (4-h delay)	34.1 ± 5.1	8.1 ± 4.4	26.0	13.8	38.2	4.46	<.001^*^
**TSD no nap participants**	**Negative scene object-background difference (M ± SE)**	**Neutral scene object-background difference (M ± SE)**	**Negative − neutral scene difference**	**95% CI lower**	**95% CI upper**	***t*-Score**	***P*-value**
Recognition 1 (10-min delay)	29.1 ± 3.3	2.8 ± 4.2	26.2	16.3	36.2	5.45	<.001^*^
Recognition 2 (4-h delay)	23.8 ± 4.5	6.0 ± .4.5	17.8	6.5	29.2	3.24	.003^*^

Within-group comparison of object-background difference scores between negative and neutral scenes within normal sleep control participants (top), TSD Nap participants (middle), and TSD No Nap participants (bottom). Object-background difference was calculated as the difference between the corrected memory score for objects and the corrected memory score for backgrounds for each valence type (negative and neutral). At both recognition sessions, each condition shows substantially greater differences in memory for the negative scene components compared to the difference in memory for the neutral scene components, demonstrating the strength of the emotional trade-off effect in both a rested and sleep deprived state. Mean ± SEM, mean difference, and 95% confidence interval (CI) reported. Within-groups comparisons done using paired-samples *t*-tests. ^*^p < .05

### Morning and afternoon self-report scores

In the morning, compared to the Sleep condition, participants in the TSD condition reported significantly reduced PA (*t*(65) = 6.5, *p* < .001, *d* = 1.68), ability to concentrate (*t*(65) = 7.28, *p* < .001, *d* = 1.89), and feelings of being refreshed (*t*(65) = 6.34, *p* < .001, *d* = 1.65), as well as significantly greater sleepiness on the SSS (*t*(65) = 7.43, *p* < .001, *d* = 1.93). The only state measure that did not significantly differ was negative affect (NA; *p* = .08). Critically, participants in the TSD_Nap_ and TSD_NoNap_ conditions did not differ on any of these measures prior to randomization (all *p*’s > .09). Also, despite having significantly lower PA at baseline, participants in the TSD_NoNap_ condition still demonstrated a significantly greater decrease in PA from evening baseline to the morning session compared to participants in the normal sleep condition (*t*(46) = 4.6, *p* < .001, *d* = 1.3).

In the afternoon session, there were again condition differences for all measures except NA (*p* = .61). Compared to the normal sleep condition, TSD_Nap_ participants continued to report significantly lower PA (*t*(40) = 2.21, *p* = .03, *d* = 0.68), and concentration (*t*(40) = 2.03, *p* = .05, *d* = 0.63), but did not differ on measures of refreshedness or sleepiness (*p*’s > .16), while TSD_NoNap_ participants continued to differ from the normal sleep condition on all metrics (PA: *t*(46) = 2.74, *p* = .01, *d* = 0.79; concentration: t(46) = 4.27, *p* < .001, d = 1.24; refreshedness: *t*(46) = 4.75, *p* < .001, *d* = 1.38, sleepiness: *t*(46) = 3.21, *p* = .002, *d* = 0.93). Compared to the TSD_NoNap_ condition, TSD_Nap_ participants reported feeling more refreshed after the 90-min nap opportunity (*t*(44) = 2.62, *p* = .01, *d* = 0.78), but did not significantly differ in self-report measures of PA, concentration, or sleepiness (*p*’s > .06).

### Correlation with nap EEG

We next conducted exploratory Spearman correlational analyses between the TSD_Nap_ behavioral findings and nap EEG to identify which sleep parameters may have contributed to the observed pre- to post-nap changes. Specifically, the primary behavioral outcomes include changes in negative object performance (i.e. *Recog_2* − *Recog_1*), including accuracy, hit rate, false alarm, and trade-off effects. Sleep metrics included conventional macrostructures (e.g. TST, SE, WASO, sleep stage proportions; see descriptives in Table 4), EEG microstructures including power spectral, spindle density, SO-spindle coupling density and strength. Two participants were excluded from this analysis due to poor EEG data quality, resulting in a final sample of 16 TSD_Nap_ participants. The only significant correlation was found between TIB and the trade-off effects for negative objects (*r* = −0.63, *p* = .009). However, this result did not survive FDR correction (*p*=.292; for correlation matrix see Supplementary Table 1).

**Table 4:** Nap EEG descriptives from TSD Nap group

Variable	Mean	SD
TIB (min)	92.81	2.01
TST (min)	88.03	5.19
SOL (min)	3.25	2.93
SE (min)	94.86	5.38
WASO (min)	2.03	3.55
N1 (min)	2.78	2.44
N2 (min)	17.66	6.81
N3 (min)	64.28	8.83
REM (min)	3.31	6.22
N1 %	3.16	2.78
N2 %	19.93	7.32
N3 %	73.02	9.23
REM %	3.88	7.23

Nap EEG descriptives. Data were obtained from 16 TSD nap participants (*n* = 2 were excluded because of poor EEG quality and sleep data was not obtained from *n* = 2 due to equipment issues). TIB, time in bed; TST, total sleep time; SE, sleep efficiency; WASO, wake after sleep onset.

## Discussion

In this study, we set out to expand upon the work of Dr. Robert Stickgold by determining the impact of TSD prior to encoding on memory for negative and neutral scene components. Additionally, we investigated the potential restorative effects of a 90-min nap opportunity memory performance. To do this, we used the well-validated ETO task, given its previously reported sensitivity to the preferential retention of emotional over neutral aspects of memory after sleep [8, 37].

Three key results from this study provide some of the strongest recent evidence for the importance of sleep in the accurate retention of emotional content over neutral information. First, as predicted, a night of TSD dramatically reduced overall memory performance, negatively impacting all scene component types (although the effect did not reach statistical significance for neutral objects). Second, the recovery nap had a greater effect than we predicted. Our second hypothesis was that the TSD_Nap_ condition would have overall better memory compared to TSD_NoNap_ condition, and given the novelty of the design and the inconsistent findings highlighted in the introduction, we did not have a specific prediction on how the individual scene components would be affected by the post-TSD recovery nap. Not only did participants in the TSD_Nap_ condition have better memory compared to TSD_NoNap_ participants at the 4-h delay, sleep deprived participants who were allowed a 90-min nap opportunity performed statistically similar to participants in the sleep control condition at the second recognition session. Moreover, when comparing memory changes between recognition sessions, memory for negative objects *improved* for TSD participants that napped; memory for negative objects was more accurate during the 4-h testing session compared to during testing after just a 10-min delay, an improvement that was driven by a decrease in the false alarm rate (i.e. identifying “new” objects as “old”) for negative objects.

### Impact of TSD and a recovery nap on emotional and neutral scene components

Current theory suggests that emotional experiences engage both the memory (hippocampus) and emotional (amygdala) centers of the brain, and the concurrent release of neuromodulators (e.g. norepinephrine) tag these memory traces as high-priority, setting them up for preferential processing during sleep [71–73]. While it has long been posited that sleep—and specifically REM sleep—drives a preferential processing of emotional information over neutral information [2, 12, 74], recent reviews and meta-analyses have called the strength and consistency of this effect into question [8–11, 14]. The results reported here, however, provide substantial support for an active role of sleep in the processing and refinement of emotional memories. While the overall negative impact of sleep deprivation during the first testing session was expected, the ability of a period of post-TSD recovery sleep to not only preserve but enhance memory accuracy specifically for negative aspects of memory exceeded our predictions. Memory performance in the Sleep control condition demonstrated that even in a rested state some deterioration occurs across periods of wakefulness, especially for neutral and peripheral aspects of memory. Unsurprisingly, all aspects of memory continued to deteriorate at a precipitous rate for sleep deprived participants that remained awake for the entire study, with only negative objects retaining any significant memory on the 4-h test. In contrast, a period of post-TSD recovery sleep led to a strikingly different trajectory in memory performance.

As noted, participants in the TSD_Nap_ condition showed no deterioration in memory performance for either neutral scene component (neutral objects and neutral backgrounds paired with neutral or negative objects) from Recog_1 to Recog_2. If this were true of negative scene components as well, it would be difficult to distinguish whether sleep led to active memory consolidation or merely provided passive protection from interference. Accurate memory for negative objects in the TSD_Nap_ condition, however, significantly *improved* from 10 min to 4 h post training. While the ETO effect has frequently been shown to increase across sleep, this significant increase in memory over time specifically for negative objects is particularly exceptional in that the effect is typically driven by a retention of memory for negative objects paired with a deterioration in memory for neutral information [26, 29, 35–38, 44, 75]. While other studies using this memory paradigm have reported a slight numerical increase in memory for the central, aversive object [37], to our knowledge, this is the first study to demonstrate a significant increase in negative object memory across an extended delay. As this improvement was largely driven by a significant decrease in false alarms at 4 h, these results indicate that sleep has an active role in specifically fine-tuning emotional aspects of our memory such that we are less susceptible to competing, false emotional information. Additional research is needed to clarify whether sleep is enhancing memory during the consolidation phase or improving retrieval abilities after a nap.

### Strength of the emotional trade-off effect

An additional unexpected outcome from this study is the validation of the strength of the ETO effect [34]. Across all three conditions and both testing sessions, the disparity in memory for negative objects compared to their paired backgrounds was always greater than the difference between the neutral scene components. Furthermore, the magnitudes of the object-background differences for negative and neutral scenes were statistically similar across all conditions for both recognition sessions. Thus, the general prioritization of negative central objects at the cost of memory for the simultaneously presented neutral peripheral details persists even in the face of extreme sleep loss, providing further validation for the utility of this task to investigate the processing of emotional and neutral aspects of memory in different contexts.

### Lack of sleep stage correlations

In line with a majority of studies that have attempted to associate preferential processing of emotional information with a particular sleep stage [14], our study failed to reveal any notable associations with typical sleep macro- and microarchitecture measures. As we only had 16 PSG recordings of high enough quality for inclusion in this exploratory analysis, the small sample size is a major limitation that may have prevented us from detecting any relationships. As noted however, we were agnostic about the potential of finding sleep stage correlations given the mixed findings discussed and the expectation that the composition of the recovery sleep period after TSD would be substantially different compared to typical overnight sleep and nap periods. Further, while the sample size reported here is likely insufficient, a larger study using the ETO task (*n* = 63) also failed to find any sleep stage correlations with emotional memory or trade-off performance [76], and even the largest sleep and emotional memory study conducted to date (*n* = 929) failed to find specific sleep stage correlations with measures of emotional memory performance [77]. In addition to the many considerations mentioned in the introduction that may be contributing to the inconsistency of these effects (e.g. type of stimuli, type of memory assessment, comparison with initial learning, type of sleep manipulation, analytic methods, etc.), there are two additional considerations we had like to highlight.

First, as computing and analytical power continues to increase and become more equitable, more researchers can go beyond the previously common sleep macroarchitecture metrics of sleep stage duration and percentage to explore different aspects of sleep microarchitecture (e.g. spectral frequencies, slow oscillations, spindle metrics, etc.) [61, 78–81]. We believe this is an important direction to continue to pursue, as attempting to determine sleep stage associations at the surface level may be a key driver of replication issues in the field. For instance, two individuals may have very similar proportions of SWS during the night but may end up on opposite ends of the correlation when slow wave activity or spindle metrics are considered.

Second, a growing number of contemporary theories suggest a collaborative relationship between sleep stages that drive the retention of most—if not all—types of memory. For instance, extrapolating work by Payne and Kensinger and Cairney et al. suggests a version of the sequential hypothesis [82–84], such that after memories are encoded and tagged during wakefulness, slow oscillation–spindle dynamics during SWS may support hippocampal-neocortical transfer of information, and the high-acetylcholine/low-norepinephrine milieu during REM sleep then offers the opportunity for further affective processing of tagged information, biasing retention of the emotionally salient aspects of memory [26, 27, 72]. This model fits our results from the TSD_nap_ group particularly well in that memory ability for all scene components was preserved across the nap, with the emotionally salient aspects receiving additional processing leading to enhanced memory performance. Sara Mednick and colleagues recently developed the REM Refining and Rescuing hypothesis that similarly suggests a complementary role between NREM and REM [85–87]. They posit that NREM sleep initially reactivates and globally downscales neural memory representations. Then, the unique neurochemical and electrophysiological blend during REM sleep acts to rescue weaker memories by increasing the signal-to-noise ratio, and multiple NREM-REM cycles further fine-tune these memory traces, permitting integration and making them more resilient to interference. While more work is needed to further support and refine these hypotheses, we believe it is prudent to continue developing theories, methods, and analyses that capture how features of multiple sleep stages may work collaboratively to shape the evolution of memories over time.

### Study implications

These findings have notable real-world implications. The ETO task has frequently been cited as a laboratory proxy for the “weapon focus effect” [88]. Comparing the trajectories in memory between the TSD_Nap_ and TSD_NoNap_ conditions suggests that sleep after witnessing an emotional event may be beneficial to both preserve the neutral details while also enhancing accuracy in memory for the emotional centers of the event. This may be an important factor when considering the veracity of eyewitness testimony after a stressful event, and merits further investigation in more ecologically-relevant scenarios.

These results also have important clinical relevance. As demonstrated, significant sleep loss can lead to poor discrimination between negative events that we did and did not experience—specifically to high endorsement of negative events that were not experienced. While this ability can be saved with recovery sleep in healthy individuals, patients with psychiatric conditions typically associated with pronounced chronic sleep disruption (e.g. depression, post-traumatic stress disorder, schizophrenia, etc.) may be prone to overestimate the number of negative experiences they have encountered. This overestimation, combined with a tendency to interpret ambiguous information as negative [89], may lead to a more negative world view overall. Again, future research will be needed to determine if improving sleep in clinical populations has direct influences on their interpretation of ambiguous information and if it might lead to a more positive perception of their day-to-day experiences.

### Limitations and future directions

The results from this study provide compelling support for sleep having an active role in the processing of emotional memories. One particular strength of this study is the lack of baseline differences between the Sleep and TSD groups (except for PA) and the lack of differences between the TSD_Nap_ and TSD_NoNap_ groups prior to the nap manipulation. Another strength is the use of the ETO paradigm to probe memory for simultaneously presented emotional and neutral content. Interestingly, while many recognition studies struggle to replicate preferential processing of emotional information (to the point that analyses from recent meta-analyses suggest free recall [9, 10]), recent reviews highlight that the ETO paradigm has “reliably replicated sleep to have a larger benefit for emotional compared to neutral items in a majority of studies” [14]. While further research is needed to distinguish the exact properties that make it such a unique task, we can speculate on a few potentially contributing factors. First, the ETO task puts neutral and emotional information in direct competition with one another WITHIN an encoding event. Most traditional emotional memory tasks that use full scenes or words do not (i.e. there is typically temporal distance between neutral and emotional content during encoding). Second, expectation of memory task can have a major impact on the outcomes of an emotional memory paradigm [9, 54], and the ETO task is more dependable at generating “incidental” encoding in that even if participants suspect or expect a subsequent memory test, no participant has ever reported expecting their memory to be tested for objects and backgrounds separately. Finally, the effects of the competition at encoding and incidental nature of the task may be further amplified by the separation of the full scenes into components during recognition to make the task substantially more difficult as participants are required to recognize objects and backgrounds outside of the context of their full scene presentation in which they were encoded. In the present study, even the Sleep control group only recognizes ~50% of the negative objects and ~30% of the neutral content after a 10-min delay. It is possible that studies that re-present the entire stimulus at recognition may have issues with ceiling effects, limiting the variability needed to detect preferential processing of emotional material during sleep. Still, it is important to note that emotional memory processing is a high-level integrative system built upon the coordinated operation of multiple lower-level perceptual, attentional, and associative processes (e.g. attention, mood, emotional perception, emotional reactivity, emotional regulation, working memory, early consolidation, discrimination, retrieval, etc.). Thus, more research is needed to determine which specific aspects of cognition are impacted by sleep loss and recovery sleep that are driving the memory effects we see here.

Despite these strengths, there are some important limitations to consider. First, while the design was intended to determine the effects of total sleep loss and recovery sleep on the processing of neutral and emotional scene components, it is unclear if these results will generalize to the less extreme sleep loss situations that are more commonly experienced. Future research could utilize sleep restriction protocols or target individuals with chronic sleep disruption to get a better sense of how these memory patterns may differ in more commonly occurring sleep loss scenarios. A second limitation is that the ETO paradigm used here exclusively used negative, aversive material as the central scene components for the emotional stimuli. It will be important to replicate this study using positive stimuli, as positive information may be affected differently when encoded in a sleep deprived state and then recalled pre- and post-recovery sleep. A third limitation is that the recognition tests took place at two different times of day. As such, it is possible that there may have been some unmeasured influence of time of day or circadian rhythm that contributed to changes from the first to second recognition task [90, 91], although this would have affected all groups equally, similar to what was observed in the PVT. A fourth limitation is that we were not able to financially afford objective sleep measurement in the sleep group and had to rely on self-report measures to estimate their overnight sleep duration. Future studies would greatly benefit from the inclusion of wearable devices for at-home monitoring at minimum to help confirm compliance and provide opportunities for additional analyses within the sleep condition. Finally, the nature of the sleep manipulations used in this study makes it difficult to fully determine what phase or phases of memory are being impacted by sleep or sleep loss. For instance, the deterioration of memory after TSD could have been driven by the effect of sleep loss on encoding, on retrieval, or a combination of the two. Similarly, the recovery nap may have affected memory performance both through active processing during early consolidation as well as leaving the participant more rested and alert during the second retrieval task. Future research should continue to develop novel study designs with the goal of isolating the effects of sleep and sleep loss on the different phases of memory processing [11].

## Conclusion

Despite these limitations, our results offer exciting new support for the active consolidation of emotional memory during sleep. As expected, TSD led to significant deterioration in memory performance along with decreases in PA, vigilance, and concentration and increases in sleepiness and fatigue. This study was also the first to investigate the impact of post-TSD recovery sleep on an emotional memory task and the findings exceeded our expectations. Not only did recovery sleep lead to improved performance compared to sleep deprived participants who remained awake, but memory performance in sleep deprived participants who napped was statistically equivalent to rested participants that slept normally the night before. Further, napping after sleep deprivation led to an enhancement in memory accuracy specifically for negative aspects of scenes. In sum, this study was able to leverage an emotional memory task that has previously demonstrated capacity to reveal sleep-dependent preferential processing of emotional stimuli to provide evidence for this effect in a novel context. Given the previously reported mixed findings [8–10], future research should continue to clarify the role that sleep plays in differential processing of emotional and neutral information in our memory and explore the underlying mechanisms generating these effects.

## Supplementary Material

OrganizedCorrelationStats_zpaf093

## Data Availability

The data underlying this article will be shared on reasonable request to the corresponding author.

## Footnotes

1 In a study from 2019, participants were either sleep-deprived or allowed to sleep normally during the early consolidation period *after* viewing the ETO images [38]. While sleep deprivation did lead to an overall impairment in memory, it did not significantly affect the difference in memory for negative objects and their paired neutral backgrounds.
